# Exploring Non-pharmacological Methods for Pre-operative Pain Management

**DOI:** 10.3389/fsurg.2022.801742

**Published:** 2022-03-04

**Authors:** Jayaditya Devpal Patil, Jessica Atef Nassef Sefen, Salim Fredericks

**Affiliations:** Royal College of Surgeons in Ireland (RCSI)-Bahrain, Al Muharraq, Bahrain

**Keywords:** pain, pre-operation, breathing techniques, cognitive behavioral therapy (CBT), patient education, post-operative (post op) pain, pre-operative anxiety

## Abstract

The management of pain is an essential aspect of surgical care, and pain levels in post-operative patients vary case by case. Treating postoperative pain is crucial as it leads to better outcomes and reduces risk of long term pain. While post-operative analgesics has been the mainstay of treatment, this mini-review explores an emerging concept which is preoperative pain management, with promising potential. Such interventions include educating patients on the expected pain outcomes and available pain medications. Non-pharmacological methods such as relaxation exercises have also proven to be effective after abdominal surgery, and educating patients on the existence of such methods pre-operatively encourages them to make use of available therapies. A major area of importance is the pre-operative psychological and emotional wellbeing of patients, as it is a strong predictor of pain and pain prognosis. Cognitive Behavioral Therapy can be effectively used to tackle preoperative anxiety and reduce pain levels. Hypnosis is another developing modality for decreasing stress. Lastly, long term pre-operative opioid use has been linked with higher pain scores and longer pain duration. This provides the basis on which pre-operative opioid weaning can lead to favorable post-operative pain outcomes.

While many of these methods have not been experimented on recipients of abdominal surgery in specific, it still paves the path for newer pain control strategies that can eventually be adopted for visceral surgery patients. This review points the reader and researchers to new and developing areas that hold the potential to revolutionize current established pain management guidelines.

## Introduction

Pain is the subjective perception of stimuli with several methods of management, which has always been an essential aspect of surgical care. Treating postoperative pain effectively leads to better outcomes and is also associated with a lower likelihood of long term pain, making it a priority in any surgical procedure (1). Inadequately controlled postoperative pain delays important recovery milestones, such as ambulation, activities of daily living and results in longer hospital stay (2). It is also a common cause for readmission and is associated with poor patient satisfaction and experience (3). However, despite its importance and due to its subjective nature, pain continues to be under-managed (4). While postoperative analgesics have been a mainstay of pain management and treatment, an emerging concept is placing more emphasis on the preoperative aspect of pain management, particularly, non-pharmacological approaches.

Another major area of importance is the psychological and emotional status of patients going into the operating room, as it has been shown to be a predictor of pain in the first 24 h (5). While many of these methods have not been experimented with on recipients of abdominal surgery specifically, the results can still pave the path to newer methods of pain control that can eventually be adopted for use in patients undergoing abdominal surgeries.

This mini review aims to highlight the little available literature on various preoperative methods for pain management and will focus predominantly on studies looking at non-pharmacological preoperative interventions. Although the initial objective was looking specifically at visceral surgery, due to limited literature, some orthopedic studies with relevant findings were also included. The various approaches covered by this review will include patient education, breathing techniques, pre-operative medication augmentation and cognitive behavioral therapy (CBT). This mini review aims to point readers and researchers into new areas that hold the potential to revolutionize current pain management guidelines.

## Patient Education

Patient education on pain management prior to surgery is 1 non-pharmacological approach that holds the potential to improve postoperative pain outcomes. A study conducted in 2015 on patients undergoing same day laparoscopic cholecystectomies found that patients receiving pre-op education on post-op pain they may experience — as well as medication adherence and reporting poorly controlled pain as soon as possible — reported a 10.9% reduction of severe pain during the first 24 hours post-operatively compared to those who did not receive any intervention. They also experienced fewer pain medication side effects, returned to normal activities sooner, and used more nonpharmacologic pain management methods such as cold packs and listening to music. However, the study is limited by a small sample size and limited time in providing the pre-op education (6). From a study such as this, it is evident that a low risk, high benefit intervention such as patient education is an appropriate area of pain therapy in all patients undergoing surgeries, and also introduces patients to other non-pharmacological methods of managing pain (such as cold packs and music in this study) that they might find useful and effective.

Another German study that looked at patients undergoing abdominal or vascular surgery showed patients who received pre-op education had a greater reduction in post-op pain. It also found that a negative outlook toward pre-op education among patients has some effect on post-op pain. While these results were not significant, it indicates that individual psychological coping styles may play a vital role in pre-op interventions for pain management (7).

Despite the availability of the above studies indicating a clear link, although of undetermined strength, between pre-op patient education on pain and pain levels, the PEDUCT trial, a randomized control trial focusing on post-op complications such as pneumonia, DVT, pulmonary embolism and patient-reported complications such as pain, anxiety, and depression, failed to show significant impact of pre-op education on post-op patient reported pain — however results from the trial were non-significant. This indicates the need for further research in this area given the limited literature and given that the general role of pre-op patient education still seems to have an appreciable impact on post-op pain — the matter at hand (8).

## Breathing and Relaxation Techniques

Another simple, yet effective approach to pain management is the use of breathing techniques. However, not enough attention is paid to its impact on patient perception of pain and the roles of different techniques that can be employed when attempting to reduce pain. One study looked at the effect deep and shallow breathing (DSB) techniques had on pain threshold, sympathetic conduction and negative feelings such as anger, tension and depression.

The study focused on two DSB techniques; one using a respiratory feedback task requiring higher concentration and the other using a relaxed DSB (rDSB) approach. Results showed that the patient's breathing technique decisively influenced autonomic and pain processing, particularly with the rDSB technique, which was vital in modulating sympathetic arousal and pain perception. The study also found a significant (*p* = < *0.001*) increase in the mean detection and pain thresholds when using the rDSB technique but no significant changes with attentive DSB (aDSB). This study measured sympathetic activity using skin conductance levels (SCL), which represents the electrical conductance of the skin. This varies with skin moisture levels affected by sweat production. As sweat production is governed by sympathetic stimulation, the SCL adequately reflects sympathetic activity. The results showed the mean SCL to be significantly decreased during the rDSB but not during aDSB. Both breathing techniques, however, showed similar reductions in negative feelings such as tension, anger, and depression (9). The raise in pain thresholds among patients using the relaxing breathing techniques and decrease of negative emotion, as well as total mood disturbances, in both groups, also show how under-utilized seemingly simple non-pharmacological interventions are in managing pain, and the promising results they can yield if implemented appropriately.

The variance in results between both groups however, leaves room to speculate around the presence of an association between visual presentation of, and the constant awareness of patient performance and pain perception. The patients in the aDSB group were “externally paced” using respiratory feedback tasks wherein they had to replicate ideal breathing curves shown to them, however those in rDSB did not have the same feedback or awareness of whether they were reaching their goals. This could perhaps be a source of anxiety that was counterproductive to pain management. From this we would recommend that such exercises be relaxing and meditative in nature rather than rigidly associated with specific goals that may result in frustration if not met.

Relaxation itself, without the use of breathing of techniques, has also been very promising. Ample amount of literature is available looking at the association of such techniques on pain reduction. One systematic review was conducted to see the efficacy of relaxation techniques on immediate pain relief in post abdominal surgery patients. The review included studies looking specifically at 4 of these techniques: Jaw relaxation, Benson's relaxation (patients relax their muscles and focus on breathing and repetition of a single word), Progressive Muscle Relaxation (PMR) and Systematic relaxation. Despite trials demonstrating the benefits of relaxation therapy for immediate pain relief post-abdominal surgery, the overall quality of the studies was not high, primarily due to small sample sizes and variations in individual relaxation protocols used. There is also lack of high-quality evidence supporting its routine use in practice and limited long term data. Despite this, the absence of harmful effects and minimal time required in training patients, these techniques may still be implemented to provide short-term pain relief (10).

One study looking specifically at older patients undergoing abdominal surgery investigated the effect of systematic relaxation techniques in 124 patients. This study found statistically significant differences in pain and anxiety, and in analgesic use between the patients in experimental and control groups (11). Another such study conducted on 60 patients undergoing upper abdominal surgery found relaxation exercises to have a significant effect (*z* = −*5.497; p* < *.001*) in reducing post-operative pain in 71.7% of the patients. The study, however, was limited by a small sample size (12).

## Cognitive Behavioral Therapy

Patient factors are also known to be strong predictors of recovery after surgery. Multiple mechanisms underlying the relationship between negative psychological states and surgical recovery have been proposed, including activation of the body's major physiological responses to stress (13) and a direct influence on increased pain perception by negative psychological states (14). This association was highlighted by a study that showed preoperative pain and anxiety symptoms to be significant predictors for pain intensity during the first 24 h post-surgery. Thus, preoperative cognitive behavioral interventions targeting patients' psychological status seem to hold the potential to decrease pain (15).

One mode of management is the mind-body therapy. Mind-body interventions include a range of practices and therapies aimed at facilitating the mind's capacity to affect health (16). Mind-body therapies assume a bidirectional relationship between the mind and the body, typically focusing on the relationships between the brain, mind, body, and behavior, and their effect on health and disease (17). They encourage patients to take responsibility for their own health and to become actively involved in their care and wellbeing. Mind-body therapies are also low cost since they can be administered by nurses and other health care providers, and are largely free from adverse side effects (18). A systematic review looking at the efficacy of pre-op mind-body based therapies on post-operative outcomes found very limited evidence suggesting that mindfulness can significantly reduce post op pain but these results were influenced by poor literature quality, small review study sample size and language bias as the search criteria was limited to studies published in English (19). This goes to show that further randomized trials are required before any conclusive data can be found and implemented in current practices of pain management.

One Multiple regression analysis focused on female patients undergoing breast cancer surgery showed patient psychological factors to have a significant impact on 1 week post op pain levels (*p* < *0.03*) and symptoms such as nausea (*p* < *0.05*) and fatigue (*p* < *0.03*). The analysis further suggests that patients with higher pre-op expectancies and emotional stress had greater risk of experiencing higher levels of post-op side effects. This analysis however was limited to patients undergoing breast cancer surgery and failed to take personality characteristics into account (20, 21). Whether similar results can be replicated in patients undergoing visceral surgery is up for debate, the findings from this study are interesting and may be replicated for appropriate study groups. Psychological interventions that can alter both patients expectancies and emotional distress prior to surgery may be particularly effective for controlling pain, fatigue and nausea following surgery but more studies need to be replicated in different populations to establish management.

One such intervention is hypnosis (22). Hypnosis can possibly lead to changes in subjective experience, alterations in perception, sensation, emotions and thoughts or behaviors, primarily owing to increased state of suggestibility (17). This increased state of suggestibility (patients ability to accept outside input) is brought about by creating a sense of awareness, arousal and concentration used to reduce stress and anxiety and increase relaxation (23). By decreasing the perception of the external environment (dissociation) created by the intense involvement of a central object of concentration (absorption), patients become more likely to accept outside input (24, 25). Hypnosis has the benefit of not only being effective for reducing distress but also being time efficient, which can be critical in a hectic surgical environments. One randomized trial looking at the effect of self-hypnosis relaxation techniques on post op pain medication requirement in patients undergoing first-time coronary artery bypass surgery found hypnosis to have a non-significant effect on pain medication use between patients practicing hypnosis and control patients. Patients, however, were found to be significantly more relaxed compared to the control group receiving no therapy (*p* = *0.032*). The results, however, were confined to a limited study population and discrepancies amongst technique used for patients (26).

## Pre-Operative Medication Use

The role of chronic medication use can also alter the perception of post-operative pain. One such class of medication are opioids. Long term use of opioids has been well known to alter sensitivity to pain, particularly leading to hyperalgesia. Its use prior to surgery also has an impact on post operative pain perception as patients with a history of, or current medication use, report higher pain scores and longer duration for pain resolution (27). Some studies have therefore suggested pre-operative weaning of long term opioids users off opioids (28). These approaches can help reduce post-op pain in electives surgeries and also avoid morbidity and drug abuse associated with chronic opioid use (29). Results from the American Society for Enhanced Recovery and Perioperative Quality Initiative Joint Consensus Statement on perioperative Management of Patients on preoperative opioid therapy in 2019 highlighted a study that has shown preoperative opioid reduction as a possible strategy to improve postoperative pain outcomes (30). The study in question looked at pre-operative opioid weaning in patients on chronic opioids undergoing a total knee or hip arthroplasty. The study concluded that patients who had undergone pre-op weaning have better pain outcomes. The results of this study however were limited to arthroplasty and a small sample size (31). This approach may be implemented in patients undergoing all surgeries, especially abdominal surgeries, to both decrease opioid overuse and decrease pain levels.

## Discussion

The most important takeaway message from this short review is that there is a vast potential for non-pharmacological methods in managing post-op pain that remains undiscovered and the pressing need for more research in this area. If these methods continue to prove to be as effective as these studies promise, it could radicalize the way pain is managed across the world and provide methods of pain relief that are effective, safe and with a low risk to benefit ratio. There are also interventions with many contradicting studies or insufficient/inconclusive results and further studies need to be undertaken in those areas to provide conclusive evidence that can construct new pain management guidelines. From the interventions discussed, non-pharmacological approaches in specific are favored because these approaches are cost effective, easier to perform, patient centered and avoid the numerous adverse effects posed by opioid and non-opioid analgesics. The transition of nonpharmacological pain management into abdominal surgery will significantly improve post-operative pain and its associated complications as well as the quality of life of patients suffering from refractory or long-term post-op pain after these surgeries. This mini review highlights the available data; and emphasizes its scarcity and the need for further research to provide conclusive data on the role of non-pharmacological approaches in pre-operative pain management in abdominal surgeries but maintains that a very promising connection exists.

## Author Contributions

JP: literature review and writing and editing of the paper. JS: literature review and writing of the paper. All authors contributed to the article and approved the submitted version.

## Funding

This work was supported by the Royal College of Surgeons in Ireland (RCSI)- Medical University of Bahrain will cover the cost of publication.

## Conflict of Interest

The authors declare that the research was conducted in the absence of any commercial or financial relationships that could be construed as a potential conflict of interest.

## Publisher's Note

All claims expressed in this article are solely those of the authors and do not necessarily represent those of their affiliated organizations, or those of the publisher, the editors and the reviewers. Any product that may be evaluated in this article, or claim that may be made by its manufacturer, is not guaranteed or endorsed by the publisher.
